# A neural network - based algorithm for predicting stone -free status after ESWL therapy

**DOI:** 10.1590/S1677-5538.IBJU.2016.0630

**Published:** 2017

**Authors:** Ilker Seckiner, Serap Seckiner, Haluk Sen, Omer Bayrak, Kazım Dogan, Sakip Erturhan

**Affiliations:** 1 Department of Urology, Gaziantep University, Gaziantep, Turkey;; 2 Department of Endustrial Engineering, Gaziantep University, Gaziantep, Turkey

**Keywords:** Calculi, Lithotripsy, therapy [Subheading]

## Abstract

**Objective:**

The prototype artificial neural network (ANN) model was developed using data from patients with renal stone, in order to predict stone-free status and to help in planning treatment with Extracorporeal Shock Wave Lithotripsy (ESWL) for kidney stones.

**Materials and Methods:**

Data were collected from the 203 patients including gender, single or multiple nature of the stone, location of the stone, infundibulopelvic angle primary or secondary nature of the stone, status of hydronephrosis, stone size after ESWL, age, size, skin to stone distance, stone density and creatinine, for eleven variables. Regression analysis and the ANN method were applied to predict treatment success using the same series of data.

**Results:**

Subsequently, patients were divided into three groups by neural network software, in order to implement the ANN: training group (n=139), validation group (n=32), and the test group (n=32). ANN analysis demonstrated that the prediction accuracy of the stone-free rate was 99.25% in the training group, 85.48% in the validation group, and 88.70% in the test group.

**Conclusions:**

Successful results were obtained to predict the stone-free rate, with the help of the ANN model designed by using a series of data collected from real patients in whom ESWL was implemented to help in planning treatment for kidney stones.

## INTRODUCTION

Kidney and ureteral stones are the third most commonly encountered pathologies in uro-logical practice after urinary infections and diseases of the prostate. The incidence of stones has been reported as 5-10% and they are observed three-fold more frequently in men than in women. The risk is high between the ages of 30 and 50 years, and patients with stones have been reported to experience stone development more than once throughout their lifetime. Most of the stones formed are eliminated through urination. The time period for the elimination process depends on the location and size of the stone. Spontaneous passage of ureteral stones smaller than 5mm is at a rate of about 80% (1). Extracorporeal Shock Wave Lithotripsy (SWL), ureterorenoscopy (URS), retrograde intrarenal surgery (RIRC), percutaneous nephrolithotomy (PNL), and open surgery are used to treat active stones.

Extracorporeal Shock Wave Lithotripsy (SWL): Despite the vital place of surgical treatment in the management of urinary system stones, surgical treatment has the disadvantage of reducing the patient's quality of life for a certain period of time, increases the length of hospitalization, and has a high-cost rate, thus favoring SWL (2, 3). The principle of SWL, where shock waves are directed over the stones, was first initiated in Russia in the 1950s; studies on the modality started in 1974, and it was first experimented on humans in 1980. SWL has become the first option in the management of urinary stones particularly those smaller than 2cm, since there is no need for surgery and anesthesia (3). The success rate in stones smaller than 2cm is 70-80%. Many factors have been reported to affect success including anatomic factors and age of the patient; type, opacity, and size of the stone; its location in the collecting system; and the anatomy of the kidney collecting system. In this study, we developed an algorithm which predicts the stone-free status of the patients in order to select the better treatment method and to notify patients.

The aim of this study was to develop a prototype artificial neural network (ANN) model from data obtained from real patients.

## MATERIALS AND METHODS

Patients who presented for SWL treatment at the Urology Department, between January 2013 and December 2015, were included in the study. Data were collected from 203 patients including gender, single or multiple nature of the stone, location of the stone, infundibulopelvic angle (IPA), history of SWL treatment, status of hydronephrosis, stone size after SWL, age, number of SWL session, size, skin to stone distance, stone density and creatinine, for a total of eleven variables. The first seven of these variables were categorical; the remaining six were numerical variables. Accordingly, sizes of the stones after SWL were determined as an output, and the size of the kidney stone was used to demonstrate the success rate of treatment.

Alyuda NeuroIntelligence Software randomized the training set, test set and validation set in the order of 100 (69.44%), 22 (15.28%) and 22 (15.28%) respectively.

### Artificial Neural Network

**Figure 1:**
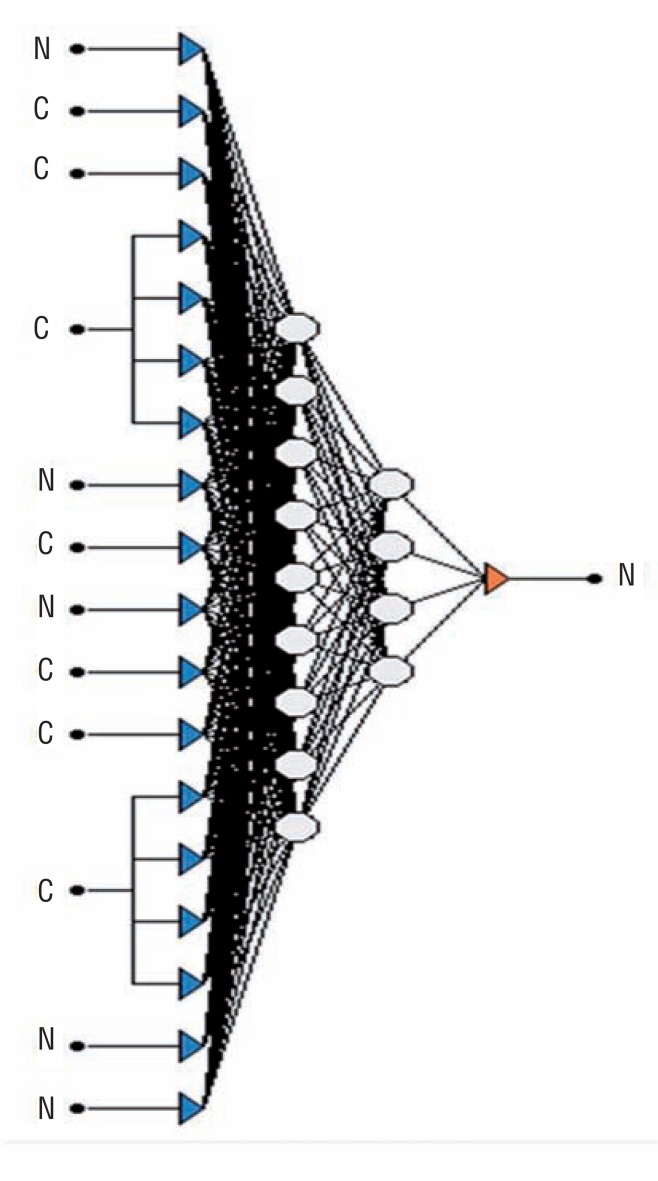
The architecture of the current algorithm.

The Alyuda NeuroIntelligence 2.2 (Alyuda Research, Inc., Los Altos, California, USA) software was used to create an artificial neural network (ANN). While creating the ANN, data were analyzed with regards to training, validity, and test. Data were analyzed according to numerical and categorical data, and also about what percentage of data was training, validity or test related. The second stage involved the transformation of all data to the numerical form for processing. In the third stage, the ANN structure was formed (Figure-1). A total of 16 neurone input values were introduced into the network structure; eight each for two intermediate neurone layers and one neurone was formed to express output. In this study, it was demonstrated that formation of two intermediate neurone layers was important to better understand the training set. The number of neurones was determined with trials. There are no particular rules in the literature expressing how to determine it. In the ANN model, all input and output values are determined according to logistic activation functions - values are transformed between the 0 and 1 interval. After determining the network structure, the training function is selected at the next training stage. This function would not normally prolong the duration of the procedure and the derivative should be an easily accessible function. Considering the features of our data, the Conjugate Gradient Descent function appeared to be the ideal training algorithm among the software programs used. Furthermore, this algorithm provided the best training means from the previous data. The classification Accuracy Ratio was considered a criterion for suspending the algorithm. This ratio was accepted as 95% for our training set. In order to prevent the duration of the training from being hindered by local minimal values, we designated the learning coefficient as 0.1 and the speed emission coefficient as 1.75. The program was designed to suspend the algorithm when the training data attained the required learning level. Weight values between the neuronal connections were constantly updated throughout the training period. An accurate prediction was said to be in place for a new case when these weights attained the most accurate value.

### Regression Analysis

The same parameters used for the ANN procedure were implemented for the regression analysis. The SPSS for Windows 22.0 (SPSS Inc, Chicago, IL) program was hence used. Significant values were calculated from coefficients obtained from the regression analysis. A value of 95% was considered significance level.

## RESULTS

A total of 203 patients were included in our study (Table-1). The patients were aged between 1 to 77 years (mean: 33.87±17.90 years). Of these patients, 121 (59.6%) were male and 82 (40.4%) were female. A hundred and thirty-eight patients (68%) were shown to have a single kidney stone, while 65 (32%) patients had multiple kidney stones. Localization of the stones in the patients was as follows: in 15 (7.4%) patients at the upper pole, in 132 (65%) patients at the middle calyx group and pelvis, in 28 (13.8%) at the lower pole, and in 28 (13.8%) patients in multiple locations. SWL was performed on 11 (5.4%) patients in one session, 25 (12.3) patients in two sessions, 24 (11.8%) patients in three sessions, 45 (22.2%) patients in four sessions, 20 (9.9%) patients in five sessions, 76 (37.4%) patients in six sessions, and on two (1.0%) patients in seven sessions. The IPA was found to be <45° in 13 (6.4%) patients, and >45° in 190 (93%) patients. The sizes of the kidney stones varied from 10-489mm^2^. They were between 0-100mm^2^ following SWL. Two hundred (98.5%) patients were receiving SWL for the first time, whereas three (1.5%) patients were receiving it for the second time. Hydronephrosis was identified in 120 (59.1%) patients; Grade 1 hydronephrosis in 39 (19.2%) patients, Grade 2 hydronephrosis in 22 (10.8%) patients, and Grade 3 hydronephrosis in 18 (8.9%) patients. There is no information about the presence of hydronephrosis in the remaining patients (2%). The creatinine levels of the patients ranged between 0.1-1.7mg/dL.

**Table 1:** Patient Characteristics.

Mean age, (range)		33.87±17.90 (1–77)
**Gender, n (%)**		
	Male	121 (59.6%)
	Female	82 (40.4)
**stone no, n (%)**		
	Single	138 (68%)
	Multiple	65 (32%)
**stone localization**		
	1 Upper pole	15 (7.4%)
	2 Middle calyx group and pelvis	132 (65%)
	3 Lower pole	28 (13.8%)
	4 Multiple locations	28 (13.8%)
**number of swL sessions**		
	1	11 (5.4%)
	2	25 (12.3%)
	3	24 (11.8%)
	4	45 (22.2%)
	5 and above	98 (48%)
**Infundibulopelvic angle**		
	<45 degree	13 (6.4%)
	>45 degree	190 (93.6%)
Mean Serum creatinine (range, mg/dL)		0.10-1.70
Pre-treatment stone size (range, mm²)		10-489
Post-SWL stone size (range, mm²)		0-100

Significant correlations were found between stone size, number of SWL session, stone location, infundibulo-pelvic angle, skin to stone distance and stone-free rate in the regression analysis (Table-2).

**Table 2:** Coefficients obtained from regression analysis.

	Standardized Coefficients	Significance
Age	-0.044	0.663
Sex	-0.107	0.291
Single/Multiple	0.268	0.007
Location	0.221	0.027
Number of sessions	0.245	0.014
IP angle	0.207	0.039
Stone size	0.394	0.001
Primary/Secondary	-0.149	0.140
Hydronephrosis	-0.019	0.848
Creatinine	0.088	0.382
Stone Density	0.056	0.578
Skin to stone distance	-0.200	0.046

Patients were randomly divided into three groups in order to implement the ANN: the training group (n=139), the validation group (n=32), and the test group (n=32).

The ANN analysis demonstrated the prediction accuracy of the stone-free rate as 99.25% in the training group, 85.48% in the validation group, and as 88.70% in the test group.

## DISCUSSION

Decision support systems (DSS), such as the ANN, can be used in the medical field, as computer generated algorithms, which help health care officials in clinical decision making. The basis of algorithms written in various forms is to mimic the learning style of the brain. These algorithms assist medical officials in decision making using specific clinical information of patients. As it is with other algorithms, real patient data should be registered in the computer. Data organization with the group of information at hand is useful, and with the help of various functions the computer is taught how to use data to make certain analysis. With the help of functions used on ANN, the computer is programed to predict certain parameters in a clever form, using training sets within the time frame. At the end of the training, the performance of the trained computer, which uses these real data is evaluated. The extent to which the computer has learned is evaluated, with regards to validity and test data. If the computer is concluded to have learned enough to predict users, the program is deemed useful.

The ANN may be very beneficial in improving the quality of health services, help in the early diagnosis of diseases, prevent medical faults, and help health officials provide appropriate treatment to patients and to reduce costs. The decision making process is terminated by the selection of one of the alternative results from cognitive processes. The cognitive process is said to increase and the possibility of making mistakes also increases with an increase in the number of alternatives. At the end of the decision, an action or an idea is formed. An investigative subject is produced in different forms to demonstrate how individuals make decisions. The interaction of psychological factors and cognitive activities with the environment should be analyzed during the decision making process. A person's mind is expected to provide certain suggestions through logical filtering, and then make the right decision by itself. Despite much research, it is not yet well understood how the human brain functions in decision making. Solid and reliable information is needed by the brain to carry out the decision making function. All alternatives should be considered together in order to make the right decision. Since knowledge is a value against time, it is necessary to provide the decision maker with the data in the shortest possible time, in order to make effective and fast decisions. As a result, many administrative and specialist organization currently require the ANN for effective and speedy decision-making.

Recently, the ANN has gained effective usage in urological practice. Most of these studies have been on diseases of the prostate, particularly on carcinoma, with acceptably successful results (4).

SWL, which has long been used in the management of urinary system stones, is especially effective in the treatment of small sized stones. However, SWL may produce poor results in a certain group of patients. From the patient's perspective, this accounts to time wasted, loss of kidney function, additional costs, and stress. As a result, it is important to know beforehand if certain treatment procedures would be successful in certain diseases. Clinicians actually use certain parameters to predict the success of treatment through discussion with their patients, and depending on their clinical experience. Studies that have been conducted to predict the success of SWL have identified certain effective parameters. However, the desired results have not been attained from these studies conducted using classical statistical methods. For example, Gomha et al. (5) used a logistic regression model to investigate stone-free status and demonstrated some significance only in the location of stones and the presence of a stent.

In the pilot study conducted by Hamid et al. (6) on 82 patients to predict the optimal fragmentation of stone on SWL, it was demonstrated that the prediction complete fragmentation was possible in a rate of approximately 75%, but reported that more advances studies are required for better results.

The current study predicted the rate of stone elimination by developing an algorithm aimed at directing the course of effective treatment, making sure that the patient received the right treatment method and providing the patient with the required information. Unlike in previous studies, our study included a larger sample size and used ANN to attain a higher stone-free rate, proving that it was possible to predict with high accuracy (99%). By so doing, data collected before treatment and registered into the system were used with an already prepared algorithm to predict the success rate of treatment. As a result, treatment modalities that were predicted as unsuccessful were discontinued in order to save cost and time, and to examine other possible treatment measures.
